# Gene transcription profiles associated with inter-modular hubs and connection distance in human functional magnetic resonance imaging networks

**DOI:** 10.1098/rstb.2015.0362

**Published:** 2016-10-05

**Authors:** Petra E. Vértes, Timothy Rittman, Kirstie J. Whitaker, Rafael Romero-Garcia, František Váša, Manfred G. Kitzbichler, Konrad Wagstyl, Peter Fonagy, Raymond J. Dolan, Peter B. Jones, Ian M. Goodyer, the NSPN Consortium, Edward T. Bullmore

**Affiliations:** 1 Department of Psychiatry, University of Cambridge, Cambridge CB2 0SZ, UK; 2 Department of Clinical Neurosciences, University of Cambridge, Cambridge CB2 0SZ, UK; 3 MRC/Wellcome Trust Behavioural and Clinical Neuroscience Institute, University of Cambridge, Cambridge CB2 0SZ, UK; 4 Research Department of Clinical, Educational and Health Psychology, University College London, London WC1E 6BT, UK; 5 Wellcome Trust Centre for Neuroimaging, UCL Institute of Neurology, University College London, London WC1N 3BG, UK; 6 Max Planck UCL Centre for Computational Psychiatry and Ageing Research, London WC1B 5EH, UK; 7 Cambridgeshire and Peterborough NHS Foundation Trust, Huntingdon PE29 3RJ, UK; 8 Immuno-Psychiatry, Immuno-Inflammation Therapeutic Area Unit, GlaxoSmithKline R&D, Stevenage SG1 2NY, UK

**Keywords:** economy, graph theory, hub, Allen Institute for Brain Sciences, transcriptome, community structure

## Abstract

Human functional magnetic resonance imaging (fMRI) brain networks have a complex topology comprising integrative components, e.g. long-distance inter-modular edges, that are theoretically associated with higher biological cost. Here, we estimated intra-modular degree, inter-modular degree and connection distance for each of 285 cortical nodes in multi-echo fMRI data from 38 healthy adults. We used the multivariate technique of partial least squares (PLS) to reduce the dimensionality of the relationships between these three nodal network parameters and prior microarray data on regional expression of 20 737 genes. The first PLS component defined a transcriptional profile associated with high intra-modular degree and short connection distance, whereas the second PLS component was associated with high inter-modular degree and long connection distance. Nodes in superior and lateral cortex with high inter-modular degree and long connection distance had local transcriptional profiles enriched for oxidative metabolism and mitochondria, and for genes specific to supragranular layers of human cortex. In contrast, primary and secondary sensory cortical nodes in posterior cortex with high intra-modular degree and short connection distance had transcriptional profiles enriched for RNA translation and nuclear components. We conclude that, as predicted, topologically integrative hubs, mediating long-distance connections between modules, are more costly in terms of mitochondrial glucose metabolism.

This article is part of the themed issue ‘Interpreting BOLD: a dialogue between cognitive and cellular neuroscience’.

## 1. Introduction

In many ways, functional magnetic resonance imaging (fMRI) is misaligned to the elementary scale of basic neuroscience. The neuron is the anatomical unit of nervous systems and the action potential is the physiological unit of communication between neurons. Functional MRI is incapable of resolving cellular structures and processes on these microscopic scales of space and time in humans. A single fMRI voxel (around 1 cubic millimetre) represents approximately 1–2 million neurons and other cells. The sampling rate of fMRI (approx. 1/60 = 0.02 Hz) is too slow to resolve rapid transients like action potentials, or the full spectrum of electrophysiological oscillations (0.1–1000 Hz). An fMRI time series will typically only resolve very low-frequency oscillations (approx. 0.1 Hz) and these ‘resting-state’ dynamics will represent local blood oxygenation changes coupled to neuronal activity rather than a direct measure of neuronal electrophysiology.

The mechanistic links between the blood oxygen-level dependent (BOLD) contrast measured by fMRI and underlying neuronal states of excitation and inhibition have been extensively investigated [1]. There is, for example, evidence that BOLD oscillations represent a hyperaemic response that is coupled to modulations in the amplitude envelope of higher frequency oscillations in local field potentials [2]. However, there remains a need for clearer explanatory connections between the molecular and cellular scale of neurovascular mechanisms of BOLD contrast in animals or other experimental models, and the whole-brain systems scale of cognitive and clinical neuroscientists using BOLD contrast for fMRI. Deeper biomechanistic interpretation of fMRI results from human experiments will inevitably be challenged by operational and ethical limits on what else can be measured in humans in an effort to control or explain the neurovascular factors of the BOLD signal. There are also a number of significant technical drawbacks to consider. BOLD contrast is not measured in SI units. A ‘raw’ human fMRI dataset is typically more noise than signal, and it can be challenging to control common and potentially severe sources of noise, such as transient micro-movements of the head (approx. 1 mm) during scanning [3,4].

To set against this list of arguments against fMRI, there are also two important advantages to consider. First and foremost, fMRI is remarkably safe and accessible for human participants including patients. Second, despite the caveats about its physiological origins, fMRI has turned out to be a highly reliable and plausible marker of local or regional brain (de-)activation by diverse cognitive tasks [5], as well as a robust signal of abnormal brain function in patients with clinical disorders [6].

In this context, it has been exciting to see new opportunities recently emerging to link fMRI phenomena to the genomic substrates of human brain organization. A pivotal role has been played by the Allen Institute for Brain Science (AIBS), which has measured expression of all approximately 20 000 genes in the human genome at each of approximately 500 locations in six post-mortem adult human brains, and publically released these data [7]. On this basis, it has been shown that human brain regions can be differentiated in terms of their transcriptional profiles of gene expression, e.g. cortex, cerebellum and thalamus have markedly different transcripts from each other. Conversely, brain regions can be aggregated with each other to constitute modules that demonstrate high co-expression of genes [7]. Gene expression profiles can also be mapped to the same anatomical space as human imaging data, enabling the first direct explorations of how the molecular mechanisms of whole-genome transcription might be related to fMRI dynamics and connectivity in humans [7,8]. For example, genes were more strongly co-expressed by functionally connected brain regions [9] and, more specifically, different classes of functional networks were distinguished by differing co-expression patterns in a set of 19 genes known to be enriched in human supragranular cortex [10]. We were motivated by these and other results to explore genomic associations with functional MRI network parameters.

Human fMRI research is arguably negotiating a shift from an interventional paradigm, focused on local signal changes estimated in response to experimentally controlled changes of cognitive state, to a more naturalistic paradigm focused on estimating very low-frequency oscillations and their correlation or functional connectivity between pairs of brain regions or voxels [11]. Functional MRI connectivity has been analysed in many different ways, ranging from simple correlational analysis, through multivariate methods such as independent component analysis (ICA), to graph theoretical analysis of the topological properties of brain functional networks [12,13]. This work has discovered that there are spatially extensive systems or networks of correlated BOLD oscillation in the human brain. These functional networks representing anatomical patterns of fMRI time-series covariance are reliable [14], heritable [15], electrophysiologically explicable in terms of amplitude envelope coupling of underlying neuronal oscillations [2], related to normal cognitive functions [16] and implicated in the pathophysiology of many clinical disorders [17].

The complex topology of human fMRI networks—comprising small-worldness, hubs and modules among other non-random and non-regular features—has been accounted for theoretically by an economical model of competition between selection pressures for both low biological cost and high topological integration [18,19]. Low cost is certainly advantageous given inevitable constraints on the intra-cranial space, biological material and metabolic resources, available to build and sustain a human brain network [20]. Topological integration is thought to be advantageous because integrated networks or global workspaces are behaviourally valuable by conferring computational capacity for adaptive, ‘higher order’ cognitive functions [18,19,21]. However, network integration is typically expensive [22]. An integrated brain network with short topological paths between all possible pairs of spatially distributed nodes will cost more in terms of the length of axonal ‘wiring’ needed to connect network nodes over long anatomical distances. The wiring cost of brain networks is nearly minimized by the anatomical co-location of densely inter-connected clusters and modules of cortical areas that typically share a specialized information-processing function [23,24]. Modularity is biologically cost-saving: but if the network is not to decompose into isolated modules, and therefore to lose capacity for global integration, there must be some connections between nodes in different modules [23]. And these inter-modular edges will generally be longer distance (higher wiring cost) than the shorter distance intra-modular edges between anatomically concentrated nodes in the same module [24,25].

On this basis, we predicted that human fMRI network nodes with high inter-modular degree, mediating many long-distance connections between modules, would be associated with a distinctive gene expression profile compared to the transcriptional profile of nodes with high intra-modular degree, mediating many short-distance connections within the same module. To test this, we measured inter-modular degree, intra-modular degree and connection distance, for each of 285 regional nodes, by graph theoretical analysis of resting-state, multi-echo echoplanar imaging data on 38 healthy young adults. We anatomically matched the fMRI network parameters at each node to detailed human brain maps of whole-genome expression provided by the AIBS (see the electronic supplementary material). Then, we used the multivariate technique of partial least squares (PLS) to identify combinations of approximately 20 000 genes whose regional expression profiles best predicted the fMRI network parameters [26]. Finally, we used a suite of recently developed gene enrichment algorithms to interpret the biological functions of genes relatively over- or under-expressed in association with specific network features [27–29].

## 2. Material and methods

### (a) Sample, functional magnetic resonance imaging data and pre-processing

In total, 2500 healthy young people in the age range 14–24 years were recruited in north London and Cambridgeshire and provided details by postal questionnaire on socio-demographics and mental health. This primary cohort was stratified into five contiguous age-related strata: 14–15 years inclusive, 16–17 years, 18–19 years, 20–21 years and 22–24 years. Recruitment within each stratum was evenly balanced for sex and ethnicity. A demographically balanced cohort (*n* = 300) was sub-sampled from the primary cohort for structural and functional MRI assessments and more detailed cognitive testing. Here, we used fMRI data from 40 participants sampled from the top two age strata of the secondary cohort (20–24 years), with 10 men and 10 women in each of the two strata. Participants were excluded if they were currently being treated for a psychiatric disorder or for drug or alcohol dependence; had a current or past history of neurological disorders including epilepsy or head injury causing loss of consciousness; had a learning disability requiring specialist educational support and/or medical treatment; or had a safety contraindication prohibiting MRI.

MRI scanning was conducted at the following three sites: (i) the Wellcome Trust Centre for Neuroimaging, London, (ii) the Wolfson Brain Imaging Centre, Cambridge, and (iii) the Medical Research Council Cognition and Brain Sciences Unit, Cambridge. All sites were identically operating 3 T whole-body MRI systems (Magnetom TIM Trio, Siemens Healthcare, Erlangen, Germany; VB17 software version) with standard 32-channel radio-frequency (RF) receive head coil and RF body coil for transmission.

Resting-state fMRI data were acquired using a multi-echo echoplanar imaging sequence with online reconstruction [30]: repetition time (TR) = 2.42 s; GRAPPA with acceleration factor = 2; flip angle = 90°; matrix size = 64 × 64 × 34; FOV = 240 × 240 mm; in-plane resolution = 3.75 × 3.75 mm; slice thickness = 3.75 mm with 10% gap, sequential slice acquisition, 34 oblique slices; bandwidth = 2368 Hz/pixel; echo times (TE) = 13, 30.55 and 48.1 ms. For pre-processing of these data, we used multi-echo independent component analysis (ME-ICA) [3,30] to identify the sources of variance in the fMRI time series that scaled linearly with TE and could therefore be confidently regarded as representing BOLD contrast. Other sources of fMRI variance, such as head movement, which were not BOLD dependent, and therefore did not scale with TE, were identified by ME-ICA and discarded. The retained independent components, representing BOLD contrast, were optimally recomposed to generate a broadband denoised fMRI time series at each voxel [3]. We used a wavelet transform for estimating functional connectivity in these data because of prior evidence indicating that cortical fMRI time series often have slowly decaying positive autocorrelation [31,32]. This approach also allowed us to focus on functional associations between brain regions based on a physiologically relevant frequency range or wavelet scale. We used a discrete wavelet transform (Daubechies 4 wavelet), resulting in a BOLD signal oscillating in the frequency range 0.025–0.111 Hz (scales 2 and 3) [33].

Pre-processing and ME-ICA was performed with the AFNI tool meica.py [3] which we slightly modified for a more stable ICA and more conservative component selection. The forked release is based on the original ME-ICA V2.5 and was released on GitHub (doi://10.5281/zenodo.50505). Wavelet decompositions were implemented using an open source, R-based software library: brainwaver v. 1.6, which is freely downloadable at: https://cran.r-project.org/web/packages/brainwaver/index.html

### (b) Functional magnetic resonance imaging connectivity and network analysis

To define regional nodes or parcels of cortex for network analysis, we used a backtracking algorithm [34] to parcellate the Freesurfer average (fsaverage) brain, subdividing regions of the Desikan–Killiany surface-based anatomical atlas of the human brain [35] into 308 smaller contiguous regions (nodes) with approximately homogeneous sizes (500 mm^2^ on the surface). This parcellation template image in standard space was transformed to the native space of each individual's fMRI dataset and regional BOLD time series were estimated by averaging the time series over all voxels in each of the 308 parcels. Some regions (particularly near the frontal and temporal poles) were excluded because of low regional mean signal in at least one subject, and two participants were excluded because of poor co-registration between functional and anatomical data. Two further regions were later excluded from the analysis due to outlier values of the corresponding gene expression data (see the electronic supplementary material, figure S1). The fMRI dataset available for analysis thus consisted of 38 individual matrices of regional mean BOLD oscillations at each of 285 cortical regions.

Functional connectivity was estimated by the pairwise wavelet correlations between each possible pair of regional mean fMRI time series. The resulting functional connectivity or association matrices were thresholded to construct binary (undirected and unweighted) adjacency matrices or graphs [36]. We used the minimum spanning tree to ensure that the graphs were node-connected even at the sparsest connection density [37]. Additional edges were then superimposed in order of decreasing inter-regional correlation to construct networks with arbitrary connection density, in the range 0–100% of the total number of possible pairwise connections.

We used standard network metrics to characterize the topology of the fMRI network (see the electronic supplementary material). To estimate the inter- and intra-modular degree of each node, we first had to define the modular community structure of each network. The modularity, *Q*(G), of a graph is proportional to the number of intra-modular edges compared to the number of intra-modular edges expected in a random graph [38]. The prototypical modules of the healthy brain functional network were derived from the sample mean wavelet correlation matrix. We used consensus modular decomposition [39] over 100 runs of the Louvain modularity algorithm on the 10% density graph constructed from this matrix. The Louvain algorithm parameter *γ* defining the coarseness of the modular partition was set at *γ* = 2, yielding eight modules. We demonstrate in the electronic supplementary material that our key results are robust to this parameter setting.

For each subject's binary graph, the degree centrality of each node, *k*(*i*), *i* = 1,2,3 … 285, was calculated as the total number of edges connecting it to the rest of the network. Total degree was subdivided into the intra-modular degree *k*_intra_(*i*), i.e. the number of edges connecting the *i*th node to other nodes within the same module, and the inter-modular degree *k*_inter_(*i*), i.e. the number of edges connecting the *i*th node to nodes in other modules. For each node, we also estimated the participation coefficient, PC(*i*), which has been widely used as a measure of nodal role in a modular community structure [40,41]:2.1
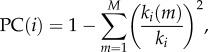
where *k_i_*(*m*) is the number of edges between node *i* and all nodes in module *m*, and the sum is over all *M* modules [42]. The PC of the index node is therefore inversely related to its intra-modular degree and will be close to PC = 0 if it is mostly connected to other nodes in the same module and close to PC = 1 if it is more homogeneously connected to all modules. The nodal measures *k*_intra_(*i*), *k*_inter_(*i*) and PC(*i*) were estimated for each subject with respect to the prototypical modular structure estimated for the sample as a whole. For each edge, we also estimated its connection distance (in millimetres) as the Euclidean distance between the linked pair of regional nodes in standard anatomical space. Topological metrics were calculated using the Brain Connectivity Toolbox, v. 2014-04-05 [43].

### (c) Microarray data and pre-processing

Microarray data for six donors (H0351.1009, H0351.1016, H0351.1015, H0351.2002, H0351.1012 and H0351.2001) were available from the AIBS (http://human.brain-map.org/static/download; [19]). Five of the donors were male and one was female; three were Caucasian, two African-American and one Hispanic; mean age = 42.5 years (see the electronic supplementary material for more information on the AIBS dataset).

We used the Maybrain package (see the electronic supplementary material) to match the centroids of the regions of the fMRI parcellation template to the closest regional gene expression profile. Microarray data were averaged across all samples from all donors in homologous regions in both hemispheres. The data were also averaged across probes corresponding to the same gene, excluding probes that were not matched to gene symbols in the AIBS database. We used the *Z*-transformation to normalize mean expression of each gene for variance in its expression (see the electronic supplementary material). The final output was a (20 737 × 285) matrix, *T*, of *Z*-scored expression values for each of 20 737 genes estimated in 285 fMRI regions. Gene expression data for individual genes or subsets, such as the 19 genes selectively over-expressed in human supragranular cortex [10], and the 162 genes specialized for aerobic glycolysis (AG) [44], were available by sub-sampling the appropriate rows of this whole-genome brain regional transcription matrix (*T*).

### (d) Parcellation into cytoarchitectonic classes

**Figure 1.**
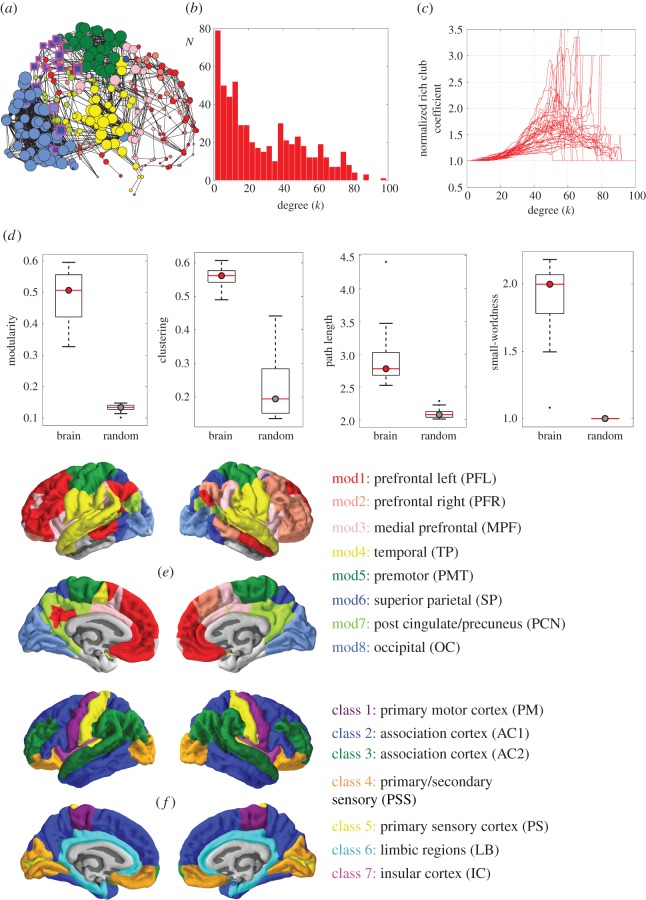
Complex topology of fMRI brain networks. (*a*) Network representation of brain functional connectivity. Colours represent eight distinct modules; the size of nodes is proportional to their degree; only the top 4% strongest connections are shown for clarity. (*b*) Degree distribution of the brain functional networks, pooled across subjects. (*c*) Normalized rich club curves of each participant's brain functional network. (*d*) Boxplots showing key network measures for the brain functional networks (in red) compared to randomized networks with preserved degree distribution (grey). From left to right, the metrics shown are: modularity Q, clustering C, path length L, and small-worldness *σ*. (*e*) Cortical surface map colour-coding brain regions according to fMRI modules, as in panel (*a*). The legend describes the approximate anatomical location of each module and defines the acronym with which each module (mod) is represented in figures 2 and 3. (*f*) Cortical surface map colour-coding brain regions according to von Economo & Koskinas's cytoarchitectonic classification [45]. Class 1 (purple): granular cortex, primary motor cortex. Classes 2 and 3 (blue and green): association cortex. Class 4 (orange): dysgranular cortex, primary/secondary sensory cortex. Class 5 (yellow): agranular cortex, primary sensory cortex. Class 6 (cyan): limbic regions, allocortex. Class 7 (magenta): insular cortex. The legend also defines the acronym with which each cytoarchitectonic class is represented in figures 2 and 3.

Each of the 308 regions in the cortical parcellation scheme was assigned to a cytoarchitectural type according to the classification scheme of von Economo & Koskinas [45] (figure 1 and electronic supplementary material, figure S2). This atlas subdivided the cortex into five types according to the laminar structure of the cortex and roughly corresponding to functional cortical specializations. Briefly, regions with poor laminar differentiation, particularly the primary motor cortex/precentral gyrus are structural type 1, regions generally considered to be association cortices are structural types 2 and 3, while secondary and primary sensory areas are types 4 and 5, respectively. The original classification of structural types does not discriminate between true six-layered isocortex and mesocortex or allocortex, which have markedly different cytoarchitectures and ontogenies [46]. We therefore defined two additional subtypes: limbic cortex which included the entorhinal, retrosplenial, presubicular and cingulate cortices, and thus primarily constitutes allocortex; and the insular cortex which contains granular, agranular and dysgranular regions, and is therefore not readily assigned a single structural type. Structural types were manually assigned to cortical regions based on visual comparison with von Economo & Koskinas's parcellation and anatomical landmarks.

### (e) Partial least squares

To explore the associations between topological centrality and distance metrics at each node of the fMRI networks, and transcription across the whole genome, we used the multivariate technique of PLS.

PLS is an established and widely used multivariate method for identifying associations between a set of response variables and a set of predictor variables, especially when the number of predictor variables exceeds the number of observations, and when the predictor variables are highly interdependent or multi-collinear [26,47]. In this case, the (285 × 20 737) regional gene transcription matrix *T* comprised the predictor variables; the (285 × 3) matrix *C* comprised the response variables of intra-modular degree, inter-modular degree and mean connection distance for each regional node of the fMRI networks.

PLS is related to principal component analysis (PCA) and combines a PCA-style dimensionality reduction with linear regression. While PCA identifies the so-called principal components in the data that best explain the variance in the predictor variables *T*, PLS finds components from *T* (gene expression) that have maximum *covariance* with the response variables in *C* (fMRI network measures). The total number of components needed to exactly fit the response *C* is limited by the number of observations (in this case the number of brain regions). The PLS components are ranked by covariance between predictor and response variables, so the first few PLS components (PLS1, PLS2, PLS3, etc.) will provide the optimal low-dimensional representation of the covariance between the higher dimensional data matrices (see the electronic supplementary material).

We used non-parametric data resampling techniques for inferential analysis of PLS results. We tested the goodness of fit of low-dimensional PLS components by repeating the analysis 1000 times after shuffling the regional labels assigned to each set of three response variables. We note that the dependency between spatially neighbouring regions that exists in both fMRI and transcriptomic datasets may lead to an over-estimation of significance by this simple, spatially naive null model. In the electronic supplementary material, figure S4, we confirm that the PLS results were robust to the use of more sophisticated null models that account for spatial correlations expected due to homogeneous tissue type within anatomical regions. We used bootstrapping (resampling with replacement of the 285 cortical regions) to estimate the error on the PLS weights estimated for each gene. The ratio of the weight of each gene to its bootstrap standard error was used to rank the genes according to their contribution to each PLS component. The list of ranked genes for the first three PLS components can be found in the electronic supplementary material: Vertes_PLS_GOenrichment.xlsx

### (f) Gene ontology and enrichment analysis

We used gene ontology (GO) tools for enrichment analysis of the ranked gene lists defined by the first three PLS components (Gorilla: http://cbl-gorilla.cs.technion.ac.il, version 30 January 2016) [27,28]. The GO terms are based on a large online database of gene annotations corresponding to ‘biological processes’ and ‘cellular components’ [48]. Enrichment analysis identified GO terms that were over-represented among the most positively and most negatively weighted genes on each PLS component. We further filtered the resulting list of enriched GO terms (i) by retaining only terms that were significantly enriched after controlling the *p*-value for significance of each term so that the false discovery rate (FDR) over all GO terms was *P*_FDR_ < 0.001 and (ii) for visualization purposes we also discarded enriched GO terms for biological processes associated with over 2500 genes, which typically correspond to general ontological terms near the top of the hierarchy, such as ‘cellular process’ or ‘organelle organization’. All excluded terms are still listed and highlighted in grey in the electronic supplementary material: Vertes_PLS_GOenrichment.xlsx.

To visualize the results of whole-genome enrichment analysis, we used the online tool REViGO (http://revigo.irb.hr) to summarize the list of significant GO terms by selecting representative subsets of the terms using a simple clustering algorithm that relies on measures of semantic similarity between terms [29]. For example, the terms ‘respiratory electron transport chain’, ‘electron transport chain’ and ‘mitochondrial electron transport, NADH to ubiquinone’ will be clustered together by the algorithm and only some of the terms will be retained. To further facilitate interpretation, REViGO was used to plot the remaining significant GO terms in semantic space, where semantically similar terms are represented close to one another. Markers are scaled according to the log_10_ of the *p*-value for the significance of each term. Clusters in these plots therefore represent families of related GO terms, and the GO term associated with the largest marker in each cluster can be annotated to label the whole cluster in a representative manner.

For the more hypothetically driven enrichment analysis of gene lists associated *a priori* with (i) supragranular layers of human cortex (human supragranular enriched, HSE) [10] or (ii) aerobic glycolysis (AG) [44] we also used permutation testing for non-parametric inference. We estimated the PLS weightings of 1000 randomly drawn sets of 19 genes and compared the PLS weights of the HSE genes to this permutation distribution to estimate the probability of HSE gene enrichment of each PLS component under the null hypothesis. We note that this permutation procedure does not take into account the correlation between HSE genes or their average expression values or their exclusively cortical origin in sampling the null distribution. More sophisticated null models for permutation testing that controlled for these or other characteristics of candidate genes will be important to develop for computational inference in future studies.

## 3. Results

### (a) Human functional magnetic resonance imaging network parameters: spatial patterning and cytoarchitectonic differentiation

We constructed graphs of the human brain functional network by binary thresholding of the pairwise inter-regional wavelet correlation (functional connectivity) matrices estimated for *n* = 38 healthy volunteers aged 20–24 years. We focus most attention on the characteristics of the graph with 10% connection density. This fMRI network had complex topological properties consistent with many prior studies [13], including a broad scale degree distribution, a rich club, a community structure comprising eight modules, high clustering, short path length and small-worldness (see figure 1*a–d* and electronic supplementary material for definitions of these widely used network metrics).

At each node in the fMRI network, we estimated four topological parameters: total degree (*k*), inter-modular degree (*k*_inter_), intra-modular degree (*k*_intra_) and PC. We note that definition of *k*_inter_, *k*_intra_ and PC will depend on both the network connection density and the resolution parameter, *γ*, defining the coarseness of the modular partition. In coarser decompositions with fewer modules, for example, fewer edges will be classed as inter-modular and the inter-modular degree distribution will shift to the left. We show in the electronic supplementary material that similar results were obtained with a coarser decomposition defining only four modules, as well as with a range of thresholds on the functional connectivity matrix, yielding networks with connection densities in the range 10–30%. For each node, we also estimated four spatial parameters: the connection distance (mean Euclidean distance of nodal edges) and the three-dimensional (*x, y, z*) coordinates of each node's location in anatomical space (electronic supplementary material, table S1).

The topological parameters were correlated with each other by construction: total degree, *k* = *k*_inter_ + *k*_intra_, was positively correlated with both inter-modular degree *k*_inter_ and intra-modular degree *k*_intra_; participation coefficient PC ∼ 1/*k*_intra_ was positively correlated with inter-modular degree and negatively correlated with intra-modular degree. The spatial parameter of connection distance was positively correlated with inter-modular degree and PC, but negatively correlated with intra-modular degree (electronic supplementary material, table S1).

**Figure 2.**
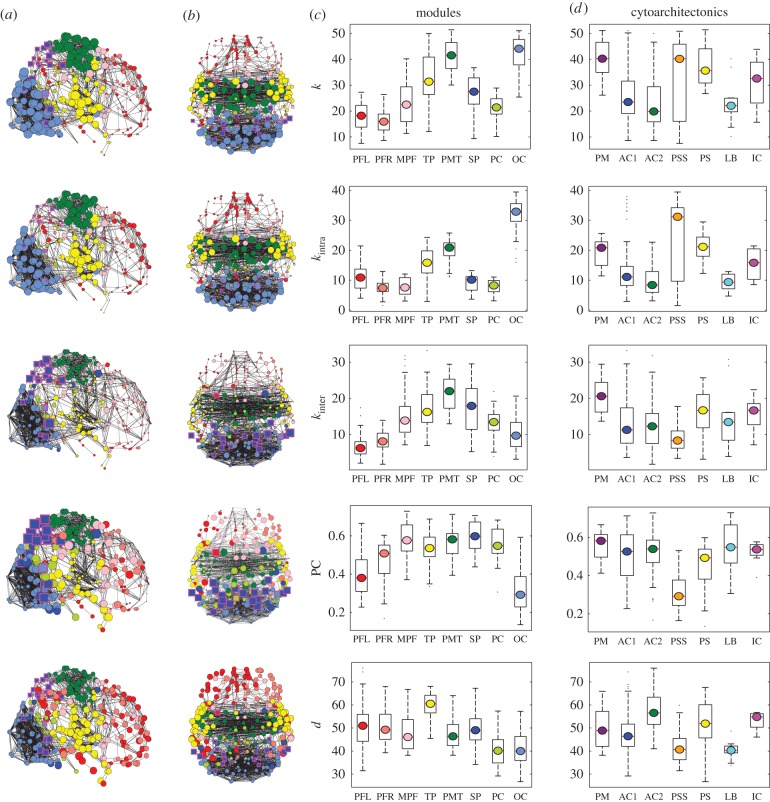
Anatomical and cytoarchitectonic patterning of fMRI network hubs. (*a*) Binary graphs constructed at 4% connection density (for clarity) showing a sagittal view of the brain. Nodal size was scaled by five nodal metrics: from top to bottom, total degree (*k*), intra-modular degree (*k*_intra_), inter-modular degree (*k*_inter_), participation coefficient (PC), and average nodal distance (*d*). Nodes are coloured by module, as defined in figure 1. Nodes with high PC (>0.5) are highlighted by square markers with a magenta outline. (*b*) Axial view of the brain networks in panel (*a*). (*c*) Boxplots showing the distribution of nodal distance and nodal topological metrics in each of the eight modules. Modules are colour-coded and named according to the scheme shown in figure 1. (*d*) Boxplots showing the distribution of nodal distance and nodal topological metrics in each of the seven cytoarchitectonic classes as defined by von Economo & Koskinas's [45] classification of cortical laminar patterns. Cytoarchitectonic classes are numbered and colour-coded according to the scheme in figure 1.

The three-dimensional location of nodes was correlated with their topological properties (table 1): intra-modular degree was negatively correlated with *y* location, meaning that intra-modular hubs were concentrated in posterior cortical regions; whereas inter-modular degree and PC were positively correlated with *z* location, meaning that inter-modular hubs were concentrated in superior cortical regions. The spatial patterning of these parameters can also be represented by anatomical maps of nodal topology and connection distance (figure 2*a*). Hubs defined by high intra-modular degree were concentrated in occipital and somatosensorimotor cortex, or spatially patterned on the rostro-caudal axis; whereas hubs defined by high inter-modular degree and PC were concentrated in somatosensorimotor and superior parietal cortex, or spatially patterned on the dorsoventral axis. Nodes with greater connection distance were concentrated in lateral cortex, reflecting the existence of long-distance, inter-hemispheric connections between bilaterally homologous cortical areas (figure 2*a–c*).

**Table 1.** Correlations between nodal fMRI variables and spatial coordinates. Matrix showing the correlations (Pearson's *r*) between MRI variables of interest as well as spatial coordinates. The *y*-coordinate corresponds to the rostro-caudal axis and the *z*-coordinate corresponds to the dorsoventral axis. We use the absolute value of the *x*-coordinate (corresponding to the medio-lateral axis) due to the symmetry between brain hemispheres. Significant correlations and anti-correlations are highlighted in italic and bold, respectively; *p* < 0.05. *k* = total degree, PC = participation coefficient, *k*_intra_ = intra-modular degree, *k*_inter_ = inter-modular degree, *d* = mean Euclidean distance of edges connecting each node to the network.

	*k*	PC	*k*_intra_	*k*_inter_	*d*	|*x*|	*y*	*z*
*k*	1.00	0.05	*0.83*	*0.63*	−0.09	−0.11	−**0.49**	*0.23*
PC	0.05	1.00	−**0.45**	*0.73*	*0.27*	*0.20*	0.05	*0.51*
*k*_intra_	*0.83*	−**0.45**	1.00	0.09	−**0.24**	−**0.19**	−**0.50**	−0.05
*k*_inter_	*0.63*	*0.73*	0.09	1.00	*0.18*	0.07	−**0.19**	*0.48*
*d*	−0.09	*0.27*	−**0.24**	*0.18*	1.00	*0.81*	*0.16*	−0.06
|*x*|	−0.11	*0.20*	−**0.19**	0.07	*0.81*	1.00	0.00	−**0.19**
*y*	−**0.49**	0.05	−**0.50**	−**0.19**	*0.16*	0.00	1.00	−0.09
*z*	*0.23*	*0.51*	−0.05	*0.48*	−0.06	−**0.19**	−0.09	1.00

Following prior work relating local cytoarchitectonic characteristics to nodal properties of human structural connectomes [49], here we explored the relationships between cortical histology and fMRI network parameters. To this end, we mapped each regional node of the fMRI network to an existing atlas of cytoarchitectonic areas classified according to the scheme of von Economo & Koskinas (figure 1*f*, §2d) [45]. Both intra- and inter-modular degree were significantly different between cytoarchitectonic classes (*k*_intra_: *F*_6,278_ = 22.7, *p* < 0.001; *k*_inter_: *F*_6,278_ = 11.1, *p* < 0.001). Intra-modular degree was highest in primary and secondary sensory cortex and lowest in association cortex and limbic regions. Inter-modular degree was highest in primary motor cortex and lowest in secondary sensory cortex. Because both total degree and PC were constructed from intra- and inter-degree, they also showed significant differences between cytoarchitectonic classes (*k*: *F*_6,278_ = 13.6, *p* < 0.001; PC: *F*_6,278_ = 22.7, *p* < 0.001; figure 2*d*), with similar patterning to *k*_intra_ and *k*_inter_, respectively. The average nodal connection distance was also significantly different between cytoarchitectonic classes (repeated measures ANOVA for main effect of class: *F*_6,278_ = 21.3, *p* < 0.001; figure 2*d*). The longest connection distances were in association cortex, whereas the shortest distances were in secondary sensory cortex and limbic regions (figure 1). We note that the association between inter-modular degree (as well as PC) and cytoarchitecture depended on the coarseness of the modular partition (see the electronic supplementary material).

### (b) Partial least-squares analysis of functional magnetic resonance imaging network parameters and gene expression

To investigate how this spatially patterned set of fMRI network nodal topology (*k*_intra_ and *k*_inter_) and distance parameters was related to local expression of approximately 20 000 genes, we co-registered the fMRI regional nodes in the same anatomical space as the AIBS dataset of human brain gene expression. For each fMRI node, we then estimated the mean regional expression of each of 20 737 genes (§2e). To explore a low-dimensional representation of the multivariate relationships between the matrix of response variables (fMRI network nodal parameters) and the matrix of predictor variables (gene expression profiles) we used PLS (§2e).

The first three PLS components accounted for about 37% of the total variance in nodal metrics and this measure of the goodness of fit was statistically significant (*p* < 0.001) for a spatially naive permutation test (see the electronic supplementary material for additional data and for spatially constrained permutation tests).

**Figure 3.**
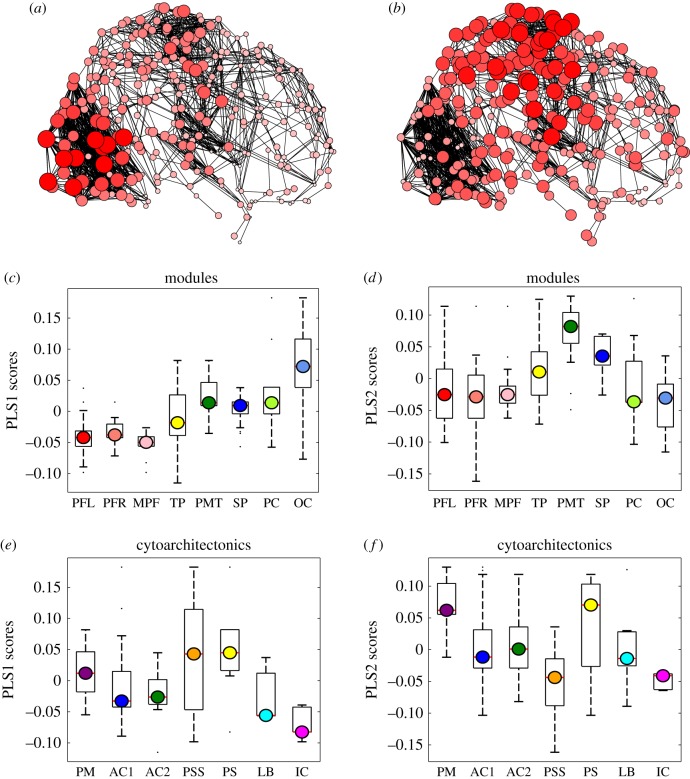
Gene expression profiles associated with fMRI network topology. (*a,b*) Binary graphs constructed at 4% connection density (for clarity) showing nodes with size and colour saturation scaled by regional scores on PLS1 (*a*) and PLS2 (*b*). Larger, darker nodes represent regions with higher PLS scores, i.e. higher expression levels of genes positively weighted on the corresponding PLS component. (*c,d*) Boxplots representing the distribution of regional scores on PLS1 (*c*) and PLS2 (*d*) in each of the eight network modules shown in figure 1. (*e,f*) Boxplots representing the distribution of regional scores on PLS1 (*e*) and PLS2 (*f*) in each of the seven cytoarchitectonic classes shown in figure 1.

The first PLS component (PLS1) was positively correlated with intra-modular degree and not significantly predictive of inter-modular degree (table 2). This means that genes positively weighted on this component were relatively over-expressed in intra-modular hubs (figures 2 and 3). Given the pattern of correlations already observed between the fMRI network parameters (table 1), it is not surprising that PLS1 was also negatively correlated with connection distance, meaning that positively weighted genes were relatively under-expressed in nodes with many long-distance connections. We also note that PLS1 results were coherent with nodal parameters (PC, *x, y, z*) that were not explicitly included in the PLS model but were known to be significantly correlated with intra-modular degree (table 1). Thus, PLS1 scores were negatively correlated with the nodal PC and were spatially patterned on the *y*-axis (rostro-caudal), with positive PLS1 scores located in posterior cortical nodes (figure 3*a,c*). Nodal PLS1 scores were also significantly different between cytoarchitectonic classes (*F*_6,278_ = 14.4, *p* < 0.001; figure 3*e*) with positive PLS1 scores located in primary and secondary sensory cortical areas.

**Table 2.** Correlations between gene expression profiles and fMRI network topology and geometry. The first three partial least-squares components (PLS1, PLS2 and PLS3) were differently correlated (Pearson's *r*) with: total degree (*k*), participation coefficient (PC), intra-modular degree (*k*_intra_), inter-modular degree (*k*_inter_,), average nodal distance (d) and spatial locations in three dimensions (|*x|, y, z*). Significant correlations and anti-correlations are highlighted in italic and bold, respectively; *p* < 0.05.

	*k*	PC	*k*_intra_	*k*_inter_	d	|*x*|	*y*	*z*
PLS1	*0.50*	−**0.26**	*0.59*	0.07	−**0.34**	−**0.19**	−**0.66**	0.10
PLS2	*0.26*	*0.45*	−0.01	*0.48*	*0.28*	*0.23*	−**0.15**	*0.53*
PLS3	0.11	−0.05	0.10	0.06	−**0.55**	−**0.67**	*0.17*	*0.15*

By contrast, the second PLS component (PLS2) was predictive of inter-modular degree and not significantly predictive of intra-modular degree (table 2). This means that genes positively weighted on this component were relatively over-expressed in inter-modular hubs (figures 2 and 3). Convergently, PLS2 was positively correlated with connection distance, meaning that positively weighted genes were relatively over-expressed in nodes with many long-distance connections. PLS2 results were coherent with nodal parameters (PC, *x, y, z*) that were not explicitly included in the PLS model but were known to be significantly correlated with inter-modular degree (table 1). Thus, PLS2 scores were positively correlated with nodal PC and spatially patterned on the *z* (dorsoventral) and *x* (medio-lateral) axes, with high PLS2 scores located in superior and lateral cortex (figure 3*b,d*). Nodal PLS2 scores were also significantly different between cytoarchitectonic classes (*F*_6,278_ = 19.4, *p* < 0.001; figure 2*f*) with the highest PLS2 scores located in primary motor and primary sensory cortical areas and the lowest scores located in secondary sensory cortex. We note that the association between PLS2 and cytoarchitecture depended on the coarseness of the modular partition because coarser partitions by definition lead to longer inter-modular connections (see the electronic supplementary material).

In short, the first two PLS components defined independent gene expression profiles that were specifically associated with (high) intra-modular degree and (short) connection distance (PLS1) or with (high) inter-modular degree and (long) connection distance (PLS2). The third PLS component defined an independent gene expression profile that was not significantly predictive of inter- or intra-modular degree (or PC), but was significantly predictive of connection distance, and was spatially patterned in all three dimensions (see the electronic supplementary material). Since we were hypothetically motivated to explore the relationships between fMRI network topology and gene expression, PLS3 was not as relevant as PLS1 or PLS2. The fourth and subsequent PLS components inevitably explained progressively smaller proportions of covariance between network metrics and gene expression, and will be less robust to noise. We therefore focus further attention only on the first two components of the PLS solution.

### (c) Enrichment analysis of gene expression profiles (PLS1 and PLS2) associated with functional magnetic resonance imaging network topology and connection distance

Given the statistical independence of PLS components, these results indicate that there are specific or distinct gene expression profiles associated with different nodal roles in fMRI networks. We used enrichment analysis to resolve the differences in transcriptional predictors of nodal topology and connection distance in more detail. We found that PLS1 and PLS2 components were significantly enriched for distinct biological functions.

**Figure 4.**
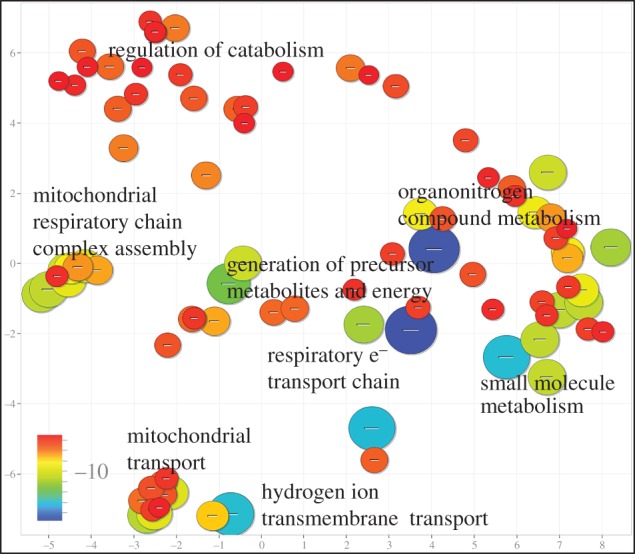
Enrichment analysis of PLS2 gene expression profile associated with long-distance connections and inter-modular hubs in fMRI networks. Significantly enriched GO terms are plotted in semantic space such that similar terms are represented close to one another. Markers are scaled and coloured according to the log_10_ of the *p*-value for the significance of each term. Large blue circles are highly significant (*P*_FDR_ < 10^−15^), while small red circles are less so (*P*_FDR_ < 0.001).

Genes significantly over-expressed in association with high intra-modular degree (positively weighted on PLS1) were significantly enriched (*P*_FDR_ < 0.001) for GO terms related to transcriptional regulation in the nucleus. By contrast, genes significantly over-expressed in association with high inter-modular degree and longer connection distance (positively weighted on PLS2) were significantly enriched (*P*_FDR_ < 0.001) for GO terms related to oxidative metabolism and mitochondria (figure 4); see the electronic supplementary material Vertes2016_PLS_GOenrichment.xlsx and figure S5 for full GO enrichment results. We additionally show in the electronic supplementary material that these results were robust to a range of parameter settings in constructing fMRI networks and to the use of alternative PLS response variables. We also show that the key results were preserved in sensitivity analyses across AIBS post-mortem brain donors.

As well as these hypothesis-free analyses that tested for enrichment of all GO terms, we also conducted two more hypothesis-driven enrichment analyses: (i) to test each PLS component for over-expression of a set of genes anatomically specific to supragranular layers of human cortex (human supragranular enriched, HSE [10]) and (ii) to test each PLS component for over-expression of a set of genes functionally specialized for aerobic glycolysis [44].

It has been shown [10] that the transcriptional profile of 19 HSE genes over-expressed specifically in supragranular layers of human cortex (but not mouse cortex) was different between brain regions with predominantly local connectivity compared to association cortical regions with a higher proportion of long-range connectivity. This motivated us to test the hypothesis that HSE genes would be differentially enriched in the first two PLS components, with greater HSE enrichment expected for PLS2 because of its specific association with long-distance and inter-modular connections in these data. As predicted, we found that HSE genes were significantly enriched among the over-expressed genes positively weighted on PLS2 (permutation test, *p* < 0.001); but HSE genes were not significantly enriched among the over-expressed genes positively weighted on PLS1.

Separately, it has been shown [44] that a set of 116 genes were functionally specialized for AG in the brain. This corresponds to non-oxidative metabolism of glucose despite the presence of oxygen. Because PLS2 was specifically enriched for oxidative metabolism genes, we were motivated to investigate whether AG genes would also be enriched in the second PLS component. We found that AG genes were not over-represented among the genes positively weighted on PLS2 (permutation test, *p* > 0.05). We note, however, that this result depended on the coarseness of the modular partition in a predictable manner, with coarser partitions by definition constraining inter-modular links to span longer distances. As connection distance was weighted more strongly on PLS2, AG genes were correspondingly more enriched in PLS2 in the analysis of the coarser four-module community structure (see the electronic supplementary material).

## 4. Discussion

The biological validation of human fMRI networks has been challenging, not least because there are fundamental questions outstanding about the biological sources of the BOLD signal itself. We cannot yet securely reduce the basic fMRI observation of inter-regional correlation between pairs of functionally connected BOLD time series to a mechanistic explanation in terms of cellular processes, neuronal physiology, neurovascular coupling or anatomical connectivity. This has made it difficult to test (refute or validate) economical models of human fMRI networks by the classical reductionist logic of explanatory mechanistic coupling between cellular processes, at a micro scale, and network topology metrics, at a macro scale (table 2).

Most of the previous evidence for biological validity of human MRI connectomes has therefore rested on analogical rather than reductionist logic. Informative analogies make comparisons between a poorly understood system and a more certainly or completely understood system of the same type. There have been several studies recently supporting the value of this approach for comparative connectomics—comparing the topology of human neuroimaging networks to the topology of more biologically specified nervous systems [50]. For example, MRI networks generally comprise a rich club of densely inter-connected high-degree hubs. Human brain network-rich clubs are topologically integrative by mediating many of the shortest paths between pairs of more peripheral nodes in different modules [51]. Rich clubs of fMRI co-activation networks are associated with diverse cognitive functions including higher order executive tasks [16], and are biologically expensive in terms of wiring cost [52,53]. These MRI observations are suggestive of an economical trade-off, between minimizing biological cost and maximizing topological value, and have been affirmed by demonstration of analogous findings in more certainly known nervous systems. In particular, the anatomical network of axonal projections and synapses between the 302 neurons of *Caenorhabditis elegans* [53] includes a topologically integrative rich club that is expensively wired and comprises command interneurons known to be functionally important for coordinated movement and adaptive behaviours [54]. Indeed, high-cost, high-value rich clubs have now been demonstrated across a wide range of scales, species, experimental techniques for network mapping, and computational models of network generation [55–58]. It seems plausible, on this basis, that the topological properties of human fMRI networks are not idiosyncratic epiphenomena but are instead representative of a general class of brain networks that have been naturally selected by the same competitive pressures for relatively low biological cost and high topological integration [18].

Here, we have provided further evidence in support of this economical model of brain network organization by a more reductionist approach. We spatially co-registered fMRI network parameters with whole-genome expression data on anatomically corresponding brain regions. We used the multivariate method of PLS to define the gene expression profiles that were optimally predictive of fMRI network parameters. We found that the first two PLS components were specifically predictive of distinct nodal network phenotypes: PLS1 was predictive of high intra-modular degree and short connection distance; whereas PLS2 was predictive of high inter-modular degree and long connection distance (PLS3 was not predictive of nodal topology; table 2). The genes most positively weighted on PLS2, and therefore relatively over-expressed in brain regions mediating many long-distance inter-modular connections, were enriched for oxidative metabolism and mitochondria. This result is clearly convergent with the expectation that integrative network features should be more metabolically expensive. It has previously been shown that hubs of human brain networks have greater rates of glucose metabolism and blood flow [59,60]; these observations have been regarded as consistent with prior knowledge of the brain's metabolic budget [61]. Synaptic transmission and maintenance of resting membrane potentials represent major demands on the brain's supply of ATP, generated mainly by mitochondrial metabolism of glucose [62]. Our data further suggest that hubs mediating more long-distance connections between modules face greater metabolic demands than hubs mediating more short-distance connections within modules. This may be because long-distance, inter-modular hubs must energetically restore and maintain electrical potentials across a greater surface area of axonal membrane. These results from human fMRI network analysis are analogous to recent results from tract-tracing and gene expression data in the mouse indicating that genes regulating oxidative metabolism were strongly co-expressed in pairs of brain regions that included a hub [63].

It was also notable that the genes associated with inter-modular degree and long distance (positively weighted on PLS2 in these data) were enriched for a set of 19 genes, expressed specifically in human supragranular cortex, that may have been necessary for the characteristically human evolution of cortico-cortical connectivity and associative cognitive processes [10]. The HSE genes were not significantly enriched in the genes associated with intra-modular degree and short distance (positively weighted on PLS1). Interestingly, the positively weighted genes on PLS2 were not significantly enriched for 116 genes specialized for AG, suggesting that the energetic resources of long-distance, inter-modular hubs are largely provided by oxidative metabolism rather than AG. However, in assessing these and other enrichment results, it is important to bear in mind that they are based on a small experimental sample (*n* = 6 post-mortem adult brains) and that individual differences in gene expression can have a marked effect on group statistics (see the electronic supplementary material for sensitivity analysis).

Interpreting the relationship between gene expression and network topology is potentially complicated by the spatial patterning of both transcriptional and topological phenotypes. Regional co-expression, or sharing of the same gene expression profile between a pair of brain regions, is related to the spatial distance between them: regions that are closer to each other will have greater genomic co-expression. Functional connectivity between regions is also conditional on distance: regions that are closer to each other are more likely to have correlated fMRI time series and so to form clusters and modules of locally inter-connected nodes in fMRI brain graphs. The question arises: does an association between gene co-expression and fMRI connectivity trivially reflect the confounding effect of distance? Previous studies have been concerned that topological clusters and modules of nodes may have transcriptional profiles in common simply because they are spatial neighbours, or members of the same cytoarchitectonic class [7]. For example, in several studies of transcriptional similarity and anatomical connectivity in the rodent brain, a statistical correction for the distance of edges was used to show that greater genomic co-expression between connected nodes was not entirely attributable to the shorter distance between them [63–68].

Our analysis of network topology has focused on nodes rather than edges and we have shown that different nodal properties have different spatial patterning, e.g. intra-modular degree was correlated with nodal location on the *y*-axis, whereas inter-modular degree was correlated with nodal location on the *z*-axis and connection distance was correlated with nodal location on the *x*-axis. PLS analysis enabled us simultaneously to explore the relationships between all three network parameters and the whole genome. We found two distinct components of gene expression that were specifically related to different aspects of nodal topology and connection distance, and had different spatial patterning as well as differential enrichment for biological processes. This pattern of results is not obviously attributable to a homogeneous and spatially isotropic effect of distance as a confounding factor of both genomic co-expression and functional connectivity [63,64,68]. Instead, these results suggest to us that multiple dimensions of spatially patterned gene expression define functionally connected systems of cytoarchitectonically similar nodes. By this account, the intertwined patterning of spatial location, nodal topology and gene expression is in fact the phenotype of interest and it would therefore not be appropriate to correct the data for spatial location prior to analysis. This is not to say that potentially problematic issues related to spatial location were entirely neglected in our analysis. For statistical inference on PLS results by permutation testing, for example, the significance of association between nodal topology and gene expression defined by the first two PLS components could be artefactually inflated by randomly permuting the regional network metrics regardless of their spatial proximity. This point is related to the more general issue of exchangeability in the proper design of permutation tests. If units of observation (regional nodes) are not expected to be independent of each other, e.g. due to their close proximity in space or time, they are not exchangeable under the null hypothesis and a permutation test based on the random permutation of individual units will not be valid. We addressed this technical concern with a block permutation algorithm that randomly permuted spatially contiguous subsets of regional nodes, rather than permuting each node individually (see the electronic supplementary material for details). This methodological refinement did not materially affect the statistical robustness of our findings although it will be important to investigate more sophisticated permutation testing methods in future.

There are a number of theoretical and methodological limitations to this work. First, the results reported here are correlational, not causative. More generally, there remains an explanatory gap in determining whether and how these specific gene profiles support short-range intra-modular connectivity (PLS1) or long-range inter-modular connectivity (PLS2). Second, the matching between fMRI and transcriptional data is imperfect as the transcriptional data are based on six human brains (mean age = 42.5 years) sampled at approximately 500 locations in each hemisphere, whereas the fMRI data are recorded from 38 healthy young adults (mean age = 22 years) sampled at 308 locations across the cerebral cortex. Third, the interpretability of enrichment analyses based on these microarray data is hampered by the fact that expression was not measured separately in different cell types. Up- or down-regulation of a gene may therefore equally represent variations in the density of certain cell types enriched for that gene or variation of expression levels of the gene within a cell type. Fourth, we approximated the distance between connected nodes by the Euclidean (straight line) distance, which is anatomically unrealistic, especially for inter-hemispheric connections between posterior parietal and anterior prefrontal cortex which follow a nonlinear trajectory constrained by the contours of the corpus callosum. In addition, our nodal measure of connection distance averaged the distance of all edges, whereas it would be informative in future studies to distinguish between the nodal distance of intra-hemispheric and inter-hemispheric or trans-callosal connections.

Nonetheless, it is encouraging to see that reductionist strategies, linking macro network properties measured in human fMRI to the underlying micro organization of cortical cytoarchitectonics and gene expression, are increasingly tractable and informative. The fMRI/mRNA results reported here have provided mechanistic support for the economic model that highly integrative network nodes, mediating many long-distance connections between modules, are metabolically expensive.

## Supplementary Material

Full GO enrichment results for PLS analysis

## Supplementary Material

Supplementary Material

## Supplementary Material

Sensitivity analysis for AIBS post mortem donors
