# Supplementary material for: Gene transcription profiles associated with inter-modular hubs and connection distance in human functional magnetic resonance imaging networks
Source: Philos Trans R Soc Lond B Biol Sci. 2016 Oct 5;371(1705):20150362. doi: 10.1098/rstb.2015.0362 (PMC5003862; doi:10.1098/rstb.2015.0362)
Supplement: Supplementary Material [file rstb20150362supp2.pdf]

DOI: 10.1098/rstb.2015.0362

Gene transcription profiles associated with inter-modular hubs and connection distance in human fMRI networks

Petra E Vértes[1], Timothy Rittman[2], Kirstie J Whitaker[1], Rafael Romero-Garcia[1], František Váša[1], Manfred G Kitzbichler[1], Konrad Wagstyl[1], Peter Fonagy[3], Raymond J Dolan[4,5], Peter B Jones[1,6], Ian M Goodyer[1,6], the NSPN Consortium[‡], Edward T Bullmore[1,6,7,8].

[1]Department of Psychiatry, University of Cambridge, Cambridge, CB2 0SZ, UK.
[2]Department of Clinical Neurosciences, University of Cambridge, Cambridge, CB2 3EB, UK.
[3]Research Department of Clinical, Educational and Health Psychology, University College London, London, WC1E 6BT, UK.
[4]Wellcome Trust Centre for Neuroimaging, UCL Institute of Neurology, University College London, London, WC1N 3BG, UK.
[5]Max Planck UCL Centre for Computational Psychiatry and Ageing Research, London, WC1B 5EH, UK.
[6]Cambridgeshire and Peterborough NHS Foundation Trust, Huntingdon, PE29 3RJ, UK.
[7]MRC/Wellcome Trust Behavioural and Clinical Neuroscience Institute, University of Cambridge, Cambridge, CB2 3EB, UK.
[8]Academic Discovery Performance Unit, GlaxoSmithKline R&D, Stevenage SG1 2NY, UK.

Contents:

1. Data accessibility, processing and QA
2. fMRI networks: metrics, modules and cytoarchitectonics
3. PLS goodness of fit and statistical significance
4. Full GO enrichment results
5. Replication of key results with coarser modular partition (4 modules)
6. Replication of key results at 5% and 15% connection density
7. Analysis with nodal distance alone as response variable
8. Robustness of results to AIBS donor data included
9. Neuroscience in Psychiatry Network (NSPN) Consortium author list

1. Data accessibility, processing and QA

All resting state data used in these analyses are on figshare:
DOI: 10.6084/m9.figshare.3363433

The file is in MATLAB .mat format and contains the following variables:

- **ROI_mni:** the x, y, z coordinates in MNI space for all 308 initial regions of interest – these are the coordinates used for matching to the Allen Institute for Brain Science gene expression data.
- **names:** the anatomical labels for all 308 initial regions of interest
- **vonEconomo:** a vector containing the cytoarchitectonic class assigned to each of the 308 cortical regions of interest. Classes are numbered 1 to 7, in the same order as defined in Figure 1 of the main text.
- **excluded_ROI_fmri:** a binary vector containing one entry per region of interest (308x1). The 21 regions which were excluded from further analysis due to fMRI dropout have value =1, all other regions have value = 0.
- **Co:** contains the 38 fully weighted 287x287 connectivity matrices for all 38 usable subjects and all 287 usable regions of interest.

- **Com:** the modular assignment of all 287 usable regions based on consensus modular partition of the group average correlation matrix at 10% connection density.
- **meas:** a structure containing nodal network metrics for all 287 usable MRI regions of interest. The measures included are: degree (meas.k), inter-modular degree (meas.Inter_k), intra-modular degree (meas.Intra_k), participation coefficient (meas.PC) and average nodal distance (meas.d).
- **excluded_ROI_genes:** a binary vector containing one entry per region of interest (308x1). The 2 regions which were excluded from PLS analyses due to outlier gene data have value =1, all other regions have value = 0.

Microarray data for 6 donors (H0351.1009, H0351.1016, H0351.1015, H0351.2002, H0351.1012, H0351.2001) are available from the Allen Institute for Brain Science (http://human.brain-map.org/static/download).

We used the allen.py function from the Maybrain package (September 2015 commit: https://github.com/rittman/maybrain/tree/11f875f0f31396c5e7adb1969c2352f2bb657748) to match the centroids of the regions of the fMRI parcellation template to the closest AIBS sample location. We then determined the anatomical region encompassing this sample, according the AIBS anatomical annotation. Microarray data were averaged across all samples from all donors in the matching anatomical region across both hemispheres. The data were also averaged across probes corresponding to the same gene (based on the AIBS matching of probes to gene symbols). We excluded probes that were not matched to gene symbols in the AIBS data. We note that we did not compare our results to alternative and potentially advantageous methods for matching probes to genes (e.g. based on Entrez IDs) and of collapsing probe-wise data by gene (e.g. using only the probe with maximum variance for each of the genes).

Finally, we used the Z-transformation to normalize mean expression of each gene for variance in its expression. This rescaling is standard for input variables to a partial least squares (PLS) analysis, as PLS ranks components (combinations of the predictor variables) by their covariance with the response variables [1]. From a biological point of view, the normalization assumes that raw expression levels do not necessarily correspond to biological effect sizes.

As described above, we excluded two MRI regions of interest (ROI) based on quality control of the gene expression data obtained in those regions. We calculated the robust range (95th percentile minus 5th percentile) characterizing the spread of gene expression data for each region of interest. Figure S1 shows the distribution of robust ranges across ROIs. The two outlier regions (range>3) in this plot correspond to:

- 'lh_lateraloccipital_part8' (occipital pole)
- 'rh_parahippocampal_part1'

These were excluded from further analysis.

The final output was a (20,737 x 285) matrix, *T*, of Z-scored expression values for each of 20,737 genes estimated in 285 fMRI regions.

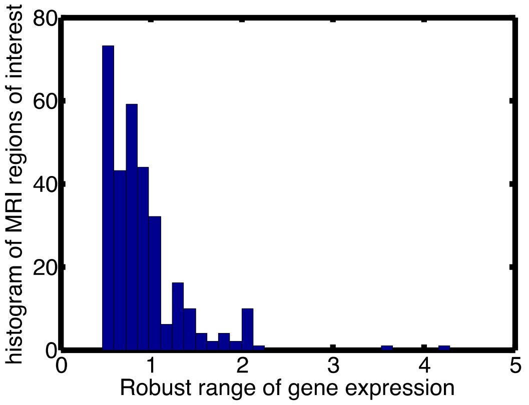

Figure S1: Histogram of the robust range of gene expression across fMRI regions of interest. Ranges above 3 were considered outliers.

2. fMRI networks: metrics, modules and cytoarchitectonics

Figure 1 in the main text illustrates that the adult fMRI networks used in this study displayed the complex topological properties expected based on prior literature. In addition to the network metrics introduced in the methods section, we used the following standard metrics to characterize the topology of the fMRI networks:

- The degree of a node is the number of other nodes it is connected to. Figure 1B in the main text shows that the brain networks have a broad scale degree distribution in which most nodes have low degree but some nodes (hubs) display high degree.

- The rich club coefficient is the ratio, for every degree k, of the number of actual versus potential edges for nodes with degree greater than k. Figure 1C shows that the hubs of the brain network connect preferentially to one another (more than expected by chance, given the degree distribution). This is measured by the normalized rich club coefficient which is above 1 for a large range of degree values, for all subjects.

- The clustering coefficient of a node is defined as the number of actual versus potential connections amongst its first neighbours. The overall clustering coefficient C is defined as the average clustering coefficient of all nodes in the graph.

- The shortest path length L_{uv} between a pair of nodes *u* and *v* is defined as the minimum number of edges that need to be traversed to get from node u to node v. The average path length, L, is the average of these values across nodes.

- The small-world coefficient (σ) is defined as the ratio of the normalized clustering (C/C_r) and path length (L/L_r) of the network, where normalization is performed with respect to the clustering (C_r) and path length (L_r) values in an equivalent random network with same degree distribution. Networks with σ >1 are considered small world as they have high levels of local clustering and short paths that globally link all nodes of the network. Figure 1D shows that the brain functional networks have higher clustering than randomized

counterparts ($C>C_r$, t-test: $P<0.01$) but that their path lengths (L) remain relatively short. This leads to the brain networks being small-world.

Figure 1 in the main text describes the anatomical location of the 8 modules identified within the fMRI data, as well as the 7 cytoarchitectonic classes previously defined [2]. Here we show, in Figure 2S, that there is no straightforward correspondence between modules and cytoarchitecture, although certain types of cytoarchitecture are over-represented in certain modules.

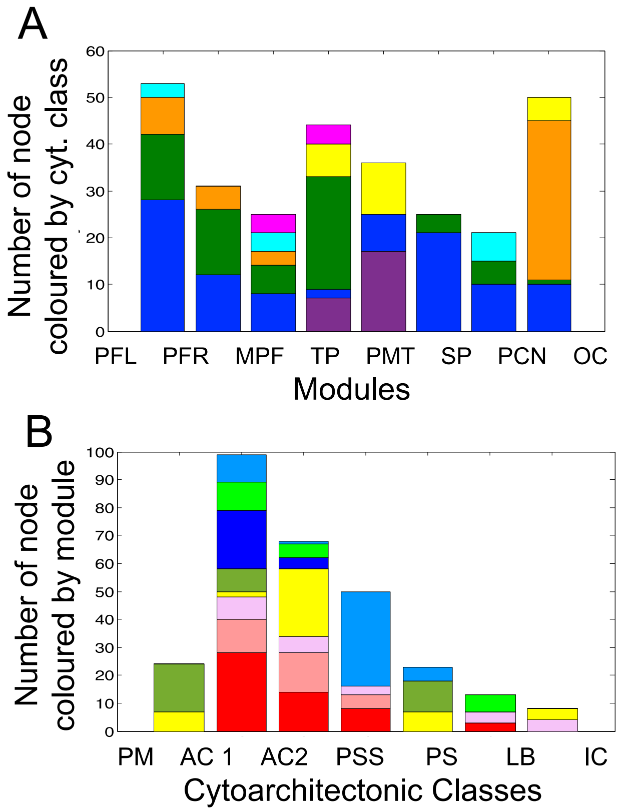

Figure S2: fMRI modules and cytoarchitectonic classes. (A) Stacked barchart showing the number of nodes from each cytoarchitectonic class (stacked segments, colour-coded as in Figure 1) falling within each module (individual bars, denoted by acronyms for modules as defined in Figure 1). (B) Stacked barchart showing the number of nodes from each module (stacked segments, colour-coded as in as in Figure 1) falling within each cytoarchitectonic class (individual bars, denoted by acronyms for cytoarchitectonic classes as defined in Figure 1).

3. PLS goodness of fit and statistical significance

Figure S3 shows the variance in response variables that is explained as a function of the number of PLS components considered. The plot does not show quick saturation as we are aiming to fit three different response variables with non-trivial correlation structure. Given that PLS components are ranked by their covariance with the response variables, we investigate them sequentially, with the aim to characterize the PLS component(s) reflecting the response pattern of interest. Amongst the first three PLS components, only PLS2 is correlated with both average nodal distance and inter-modular degree (see Table 2 in main text). In the main manuscript we

therefore focus our attention on this component, which corresponds to long-distance inter-modular connectivity.

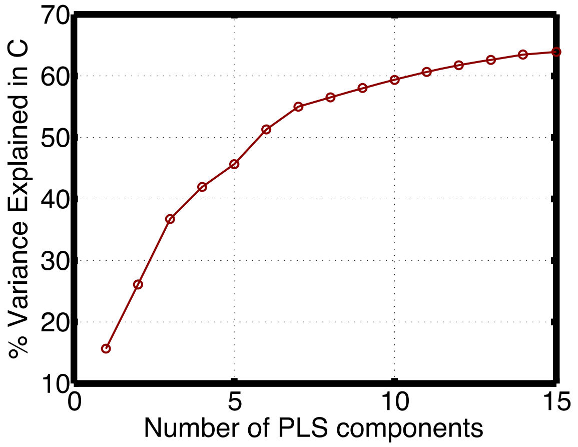

Figure S3: Variance in response variables (C) explained increases with the number of PLS components considered.

To estimate the statistical significance of the PLS solution we use a permutation test comparing the variance explained by the first two components for the observed response variables to the distribution of variance explained in 1000 analyses with permuted response variables. The permutation was done dependently, maintaining correlations across nodes within both datasets fixed, and only shuffling the assignment of regions of interest between the gene and topological matrices.

We note that the spatial structure known to exist in both fMRI and transcriptomic datasets will lead to an over-estimation of significance with this simple, spatially naïve null model. This point is related to the more general issue of exchangeability in the proper design of permutation tests. If units of observation (regional nodes) are not expected to be independent of each other, e.g., due to their close proximity in space or time, they are not exchangeable under the null hypothesis and a permutation test based on the random permutation of individual units will not be valid. We addressed this technical concern with two additional tests using more sophisticated null models that account for spatial correlations expected to manifest due to homogenous tissue type within anatomical regions. In particular, we used a block permutation algorithm that randomly permutes spatially contiguous subsets of regional nodes, rather than permuting each node individually.

In the first case we agglomerated all regions of interest (ROIs) into 61 blocks each corresponding to a distinct anatomical region as defined by the Desikan-Killiany atlas (with only 61 regions remaining due to the exclusion of regions with dropout described above). We also restricted the analysis to regions in the left hemisphere only to eliminate artefactual inflation in significance due to the symmetry of brain structures. This permutation test confirmed significance of the PLS solution (using the top two components) with $P < 0.001$.

In the second case, we explored how the P-value changes as block-size is systematically increased. We ordered ROIs in one dimension by the location of their centroid along the rostro-caudal axis (still restricting our attention to ROIs in the left hemisphere). We then repeated the block-wise permutation test with increasingly large block sizes, starting with block size = 1 (permutation ROIs individually) and

ending with block-size = 69 (yielding only two large blocks). Figure S4 shows that the PLS solution remained highly significant for large block sizes (size ~ 30).

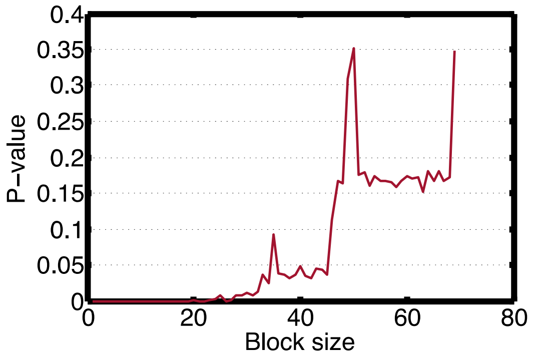

Figure S4: **Significance of PLS solution with two components as a function of block-size in the block-wise permutation test.**

4. Full GO enrichment results

Full GO enrichment results for both positive and negative PLS1 and PLS2 rankings are available in the supplementary file: **Vertes2016_PLS_GOenrichment.xlsx**

These are organized into four sheets labelled “PLS1 pos” for positive PLS1 ranking, “PLS1 neg” for negative PLS1 ranking and so on. Each sheet contains, in column A, the ranked list of genes corresponding to that PLS component. Columns C-H contain the results of enrichment using GOrilla as described in the methods section and keeping only GO terms with significance levels of $P_{FDR}<0.001$. Enriched GO terms associated with over 2500 genes were considered too general and not carried through to the REViGO analysis. These are shown as greyed out rows in the xlsx file. Column G is labelled “Enrichment (N, B, n, b)”. These values are output by the GOrilla algorithm and correspond to:

N: total number of usable annotated genes in the analysis
B: total number of genes associated with given GO term
n: number of genes in the top of the user's input list (this is the dynamically calculated threshold implemented by GOrilla)
b: number of genes in the intersection
Enrichment = (b/n) / (B/N)

Columns J-O in the **Vertes2016_PLS_GOenrichment.xlsx** file have the same structure as described above for columns C-H and correspond to the enrichment results for “cellular components” (rather than “biological processes”).

REViGO analyses use as input the list of GO terms (eg GO:0016192) and the associated FDR P-values for all terms shown in the xlsx file (except those greyed out as described above). Figure S5 shows the resulting REViGO plots for positive and negative PLS1 and PLS2 in terms of both “cellular components” and “biological processes”.

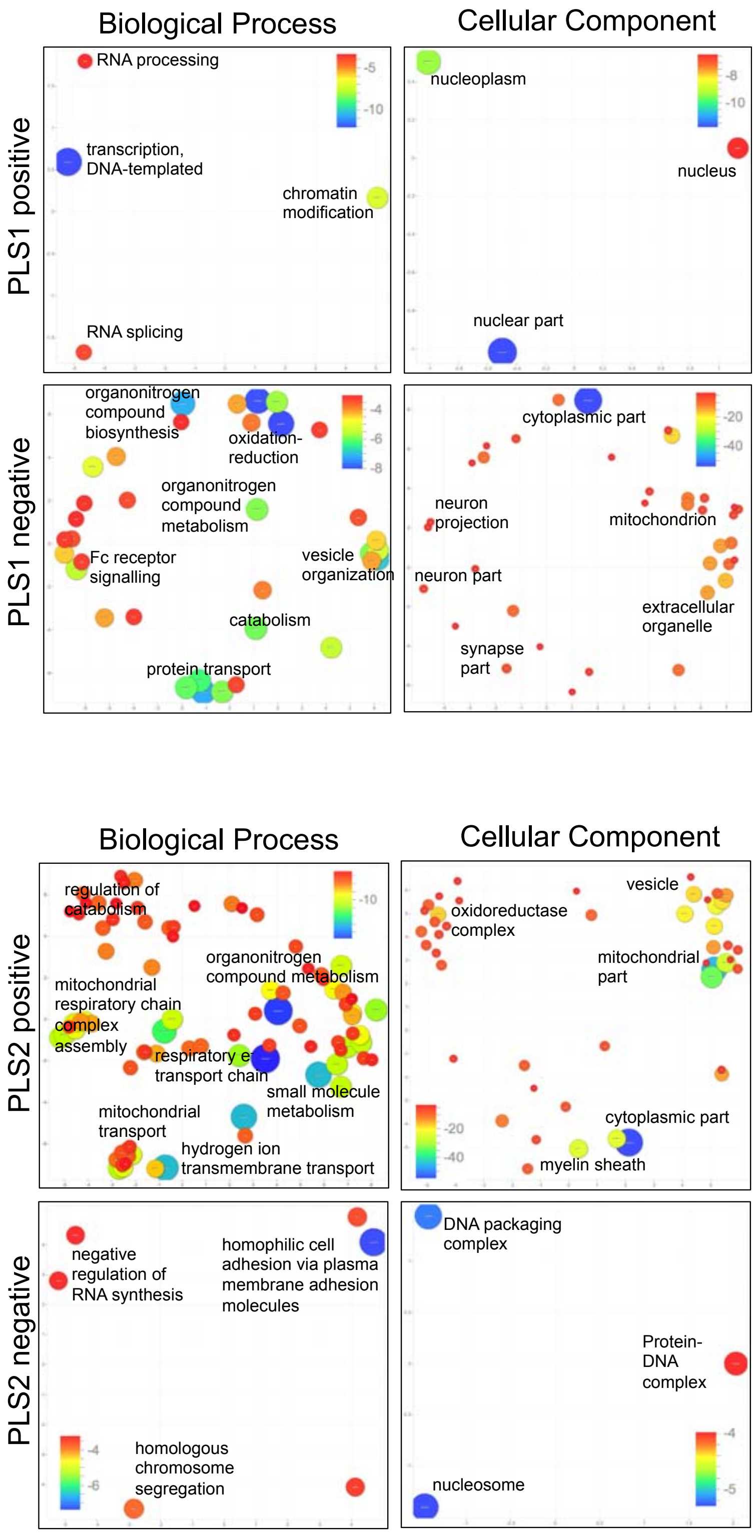

Figure S5: Full enrichment pattern for both positive and negative PLS1 (top 4 panels) and PLS2 (bottom4 panels) in terms of biological processes (left) and cellular components (right). REViGO plots showing significantly enriched GO terms in semantic space such that similar terms are represented close to one another. Markers are scaled and coloured according to the log10 of the P-value for

the significance of each term (the scale is different for each plot – for full list of values please refer to file: **Vertes2016_PLS_GOenrichment.xlsx**).

5. Replication of key results with coarser modular partition (4 modules)

Using a coarser modular partition of the fMRI network at a fixed connection density (density=10% as in main text) will lead to fewer edges being counted as inter-modular. Beyond affecting the distribution of nodal metrics amongst modules (Figure S6 A-B), this more stringent definition of inter-modularity will affect the calculation of several of the measures themselves, such as k_{intra}, k_{inter} and PC. We see indeed that the boxplots showing how these measures are distributed by cytoarchitectonic class are somewhat different in this case (Figure S6 C). In particular, nodes with high participation coefficient are now more explicitly over-represented in association areas (cytoarchitectonic classes 2 and 3) and in limbic regions (class 6).

Despite these differences, we find that the PLS results remain comparable, with PLS1 corresponding mainly to short distance intra-modular connectivity and PLS2 corresponding to long-distance inter-modular connectivity (see Figure S7). These results remained significant with block-wise permutation testing (P < 0.001 for blocks defined by the anatomical regions of Desikan-Killiany atlas, left hemisphere only).

Given the pattern of results in Figure S6, we also confirmed that PLS2 is still enriched for GO terms related to mitochondrial respiration and energy generation (Figure S8). Finally, we note that the pattern of enrichment for the two candidate gene sets is preserved: HSE genes are enriched in PLS2 (P<0.001) but AG genes are not (P>0.05).

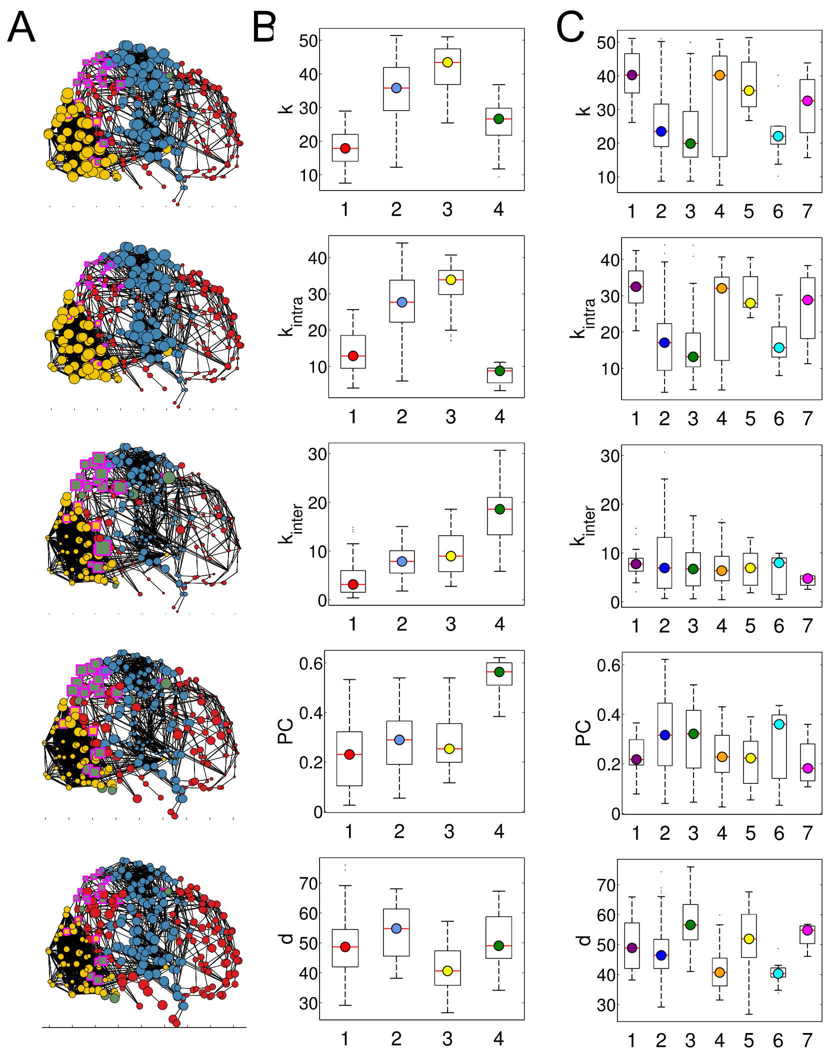

Figure S6: Anatomical and cytoarchitectonic patterning of fMRI network hubs for 4-module decomposition (γ=1). (A) Binary graphs constructed at 4% connection density (for visualization) showing a sagittal view of the brain. Nodes size was scaled by five nodal metrics: from top to bottom, total degree (k), intra-modular degree (kintra), inter-modular degree (kinter,), participation coefficient (PC) and average nodal distance (d). Nodes are coloured by module (red, frontal; blue, central; yellow, occipital; green, dorsal parietal) and nodes with high participation coefficient (PC > 0.5) are highlighted by square markers with a magenta outline. (B) Boxplots showing the distribution of nodal distance and nodal topological metrics in each of the 4 modules. Modules are colour-coded according to the same scheme as in panel A. (C) Boxplots showing the distribution of nodal distance and nodal topological metrics in each of the 7 cytoarchitectonic classes as defined by von Economo's classification of cortical regions according to cortical laminar pattern. Cytoarchitectonic classes are numbered and colour-coded according to the same scheme as in Figure 1 of the main text. Class1 (purple): granular cortex, primary motor cortex. Classes 2 and 3 (blue and green): association cortex. Class 4 (orange): dysgranular cortex, primary/secondary sensory cortex. Class 5 (yellow): agranular cortex, primary sensory cortex. Class 6 (cyan): limbic, allocortex. Class 7 (magenta): insular cortex.

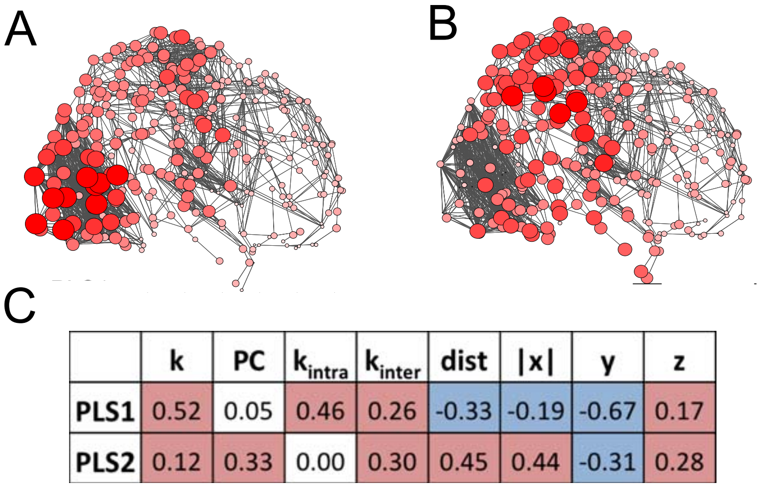

C

	k	PC	k_{intra}	k_{inter}	dist	|x|	y	z
**PLS1**	0.52	0.05	0.46	0.26	-0.33	-0.19	-0.67	0.17
**PLS2**	0.12	0.33	0.00	0.30	0.45	0.44	-0.31	0.28

Figure S7: Top two PLS components for the 4-module decomposition (γ=1). Binary graphs constructed at 4% connection density (for visualization) showing nodes with size and colour saturation scaled by regional scores on PLS1 (A) and PLS2 (B). Larger, darker nodes represent regions with higher PLS scores, i.e. higher expression levels of genes positively weighted on the corresponding PLS component. (C) Table showing how the first two partial least squares components (PLS1 and PLS2) were correlated with: total degree (k), participation coefficient (PC), intra-modular degree (k_{intra}), inter-modular degree (k_{inter},), average nodal distance (dist), and spatial locations in 3 dimensions (|*x*|, *y, z*). Significant correlations and anticorrelations are highlighted in red and blue respectively; $P < 0.05$

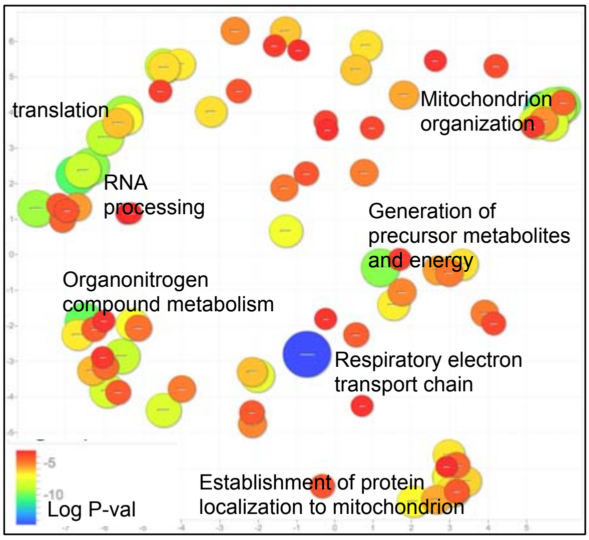

Figure S8: Enrichment analysis of gene expression profile associated with PLS2 in fMRI networks for 4-module decomposition (γ=1). Significantly enriched GO terms are plotted in semantic space such that similar terms are represented close to one another. Markers are scaled and coloured according to the log10 of the *P*-value for the significance of each term. Large blue circles are highly significant ($P_{FDR} < 10^{-15}$) while small red circles are less so ($P_{FDR} < 0.001$).

6. Replication of key results at 20% and 30% connection density

Using fMRI networks with increasing connection density will lead to different modular decompositions and different spatial distribution of nodal metrics such as k, k_{intra}, k_{inter}, PC and average nodal distance d.

For example, if we preserve the parameter setting for the coarseness of modular decompositions (γ=2, as in main text), at 30% connection density we obtain 42 modules. This includes 7 larger modules with over 20 nodes in each (reduced from 10 large modules in the case of 10% density), as well as 35 further 'modules' each including a single node. While this modular partition could be made more coherent by adjusting the parameters of the modular decomposition algorithm, here we keep parameters unchanged both for fair comparison to the results in the main text and in order to test the robustness of these results.

As might be expected due to this fragmentation of modules, we see that the distribution by cytoarchitectonic class of PC, k_{inter} and especially k_{intra} appears different in the 30% density case (Figure S9C). Despite these differences, we find that the PLS results remain comparable, with PLS2 still uniquely corresponding to long-distance hubs with increased inter-modular connectivity at both 20% and 30% connection densities (see Figure S10). Once again, results remained significant with block-wise permutation testing ($P < 0.001$ for blocks defined by the anatomical regions of Desikan-Killiany atlas, left hemisphere only).

Given the pattern of results in Figure S10, we also confirmed that PLS2 is still enriched for GO terms related to mitochondrial respiration and energy generation at both 20% and 30% density (Figure S11).

Finally, we note that the pattern of enrichment for the two candidate gene sets is preserved. For both 20% and 30% fMRI network density, HSE genes are enriched in PLS2 (P<0.001) but AG genes are not (P>0.05).

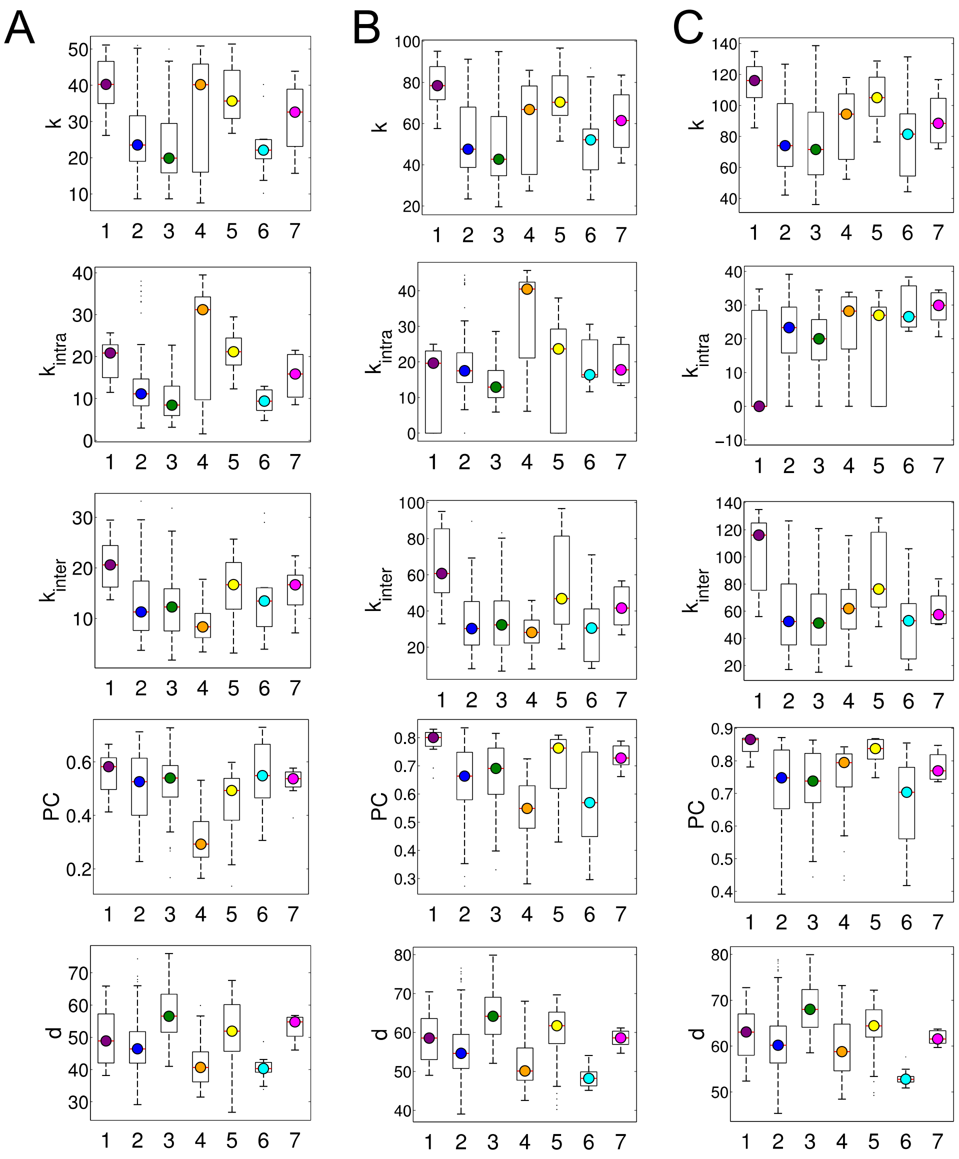

Figure S9: Cytoarchitectonic patterning of fMRI network hubs at various connection densities (all at γ=2). Boxplots showing the distribution of nodal distance and nodal topological metrics in each of the 7 cytoarchitectonic classes as defined by von Economo's classification of cortical regions according to cortical laminar pattern. Panel columns (A), (B) and (C) correspond to 10%, 20% and 30% connection density respectively. Cytoarchitectonic classes are numbered and colour-coded according to the same scheme as in Figure 1 of the main text. Class1 (purple): granular cortex, primary motor cortex. Classes 2 and 3 (blue and green): association cortex. Class 4 (orange): dysgranular cortex, primary/secondary sensory cortex. Class 5 (yellow): agranular cortex, primary sensory cortex. Class 6 (cyan): limbic, allocortex. Class 7 (magenta): insular cortex.

density=10%

	PLS1	PLS2
k	0.50	0.26
PC	-0.26	0.45
k_{intra}	0.59	-0.01
k_{inter}	0.07	0.48
dist	-0.34	0.28
|x|	-0.19	0.23
y	-0.66	-0.15
z	0.10	0.53

density=20%

	PLS1	PLS2
k	0.38	0.36
PC	-0.17	0.52
k_{intra}	0.51	-0.26
k_{inter}	0.08	0.51
dist	-0.38	0.20
|x|	-0.23	0.21
y	-0.68	-0.06
z	0.04	0.54

density=30%

	PLS1	PLS2
k	0.45	0.23
PC	0.39	0.32
k_{intra}	-0.06	-0.38
k_{inter}	0.39	0.33
dist	-0.34	0.30
|x|	-0.19	0.36
y	-0.64	0.12
z	0.26	0.40

Figure S10: Top two PLS components for fMRI network with varying density (all at γ=2). Three tables showing PLS results for increasing connection density (left to right: density = 10%, 20%, 30%). Each table shows how the first two partial least squares components (PLS1 and PLS2) were correlated with: total degree (k), participation coefficient (PC), intra-modular degree (k_{intra}), inter-modular degree (k_{inter},), average nodal distance (dist), and spatial locations in 3 dimensions (|*x*|, *y, z*). Significant correlations and anticorrelations are highlighted in red and blue respectively; $P < 0.05$

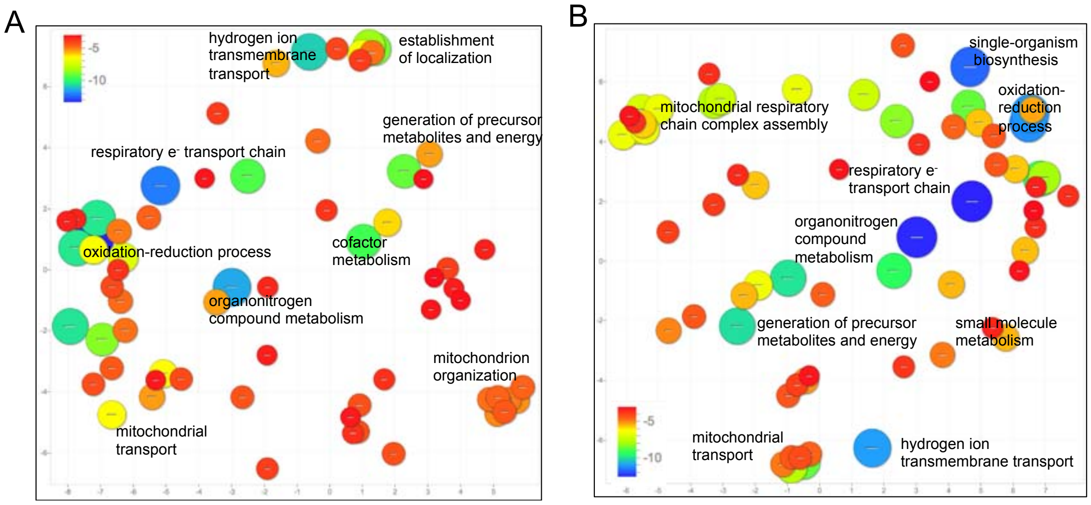

Figure S11: Enrichment analysis of gene expression profile. The two panels show the enrichment in GO biological processes associated with PLS2 in fMRI networks with 20% (A) and 30% (B) density – both with modules defined at γ =2. Significantly enriched GO terms are plotted in semantic space such that similar terms are represented close to one another. Markers are scaled and coloured according to the log10 of the *P*-value for the significance of each term. Large blue circles are highly significant while small red circles are less so. All terms shown are significant at $P_{FDR} < 0.001$.

7. Analysis with response variable including only nodal distance

Supplementary sections 5 and 6 suggest that the key results are robust to some variation in how nodal metrics are defined. In particular, we have seen that the calculation of k_{intra} and k_{inter} is sensitive to the modular decomposition (whether the latter is affected by the coarseness parameter in the decomposition itself or the density of the network). Nodal distance (d) will also be somewhat affected by the network density considered but it does not depend on modular decomposition and is thus more stable across the different analyses in supplementary sections 5 and 6.

We were therefore motivated to test whether similar results would be obtained using average nodal distance alone as a response variable. Below, we show that the key results are maintained (at density=10% and γ=2, as in main text).

Firstly, we note that the percentage of variance explained saturates much more rapidly in this case as only a single variable is to be explained (Figure S12). In particular, the top two components are sufficient to explain 50% of the variance in nodal distance.

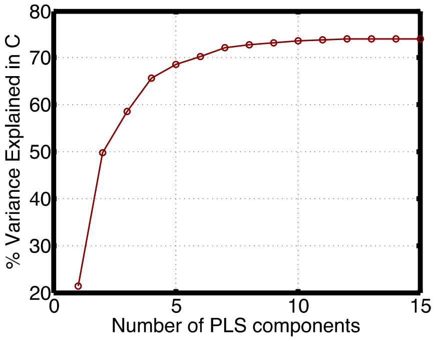

Figure S12: Percentage of variance in response variable (C = average nodal distance) explained increases with the number of PLS components considered.

Although k_{intra} and k_{inter} were not included as response variables, the PLS solution still finds two independent components (PLS1, PLS2), each explaining a large amount of variance and displaying different spatial patterning, and different relationships with k_{intra} and k_{inter} for these parameter settings. In Figure S14 we show that the GO enrichment pattern of PLS2 still corresponds to oxidative metabolism in mitochondria.

	k	PC	k_{intra}	k_{inter}	dist	|x|	y	z
**PLS1**	0.43	-0.02	0.40	0.18	-0.46	-0.33	-0.58	0.08
**PLS2**	0.17	0.22	0.08	0.22	0.53	0.63	-0.36	0.19

Figure S13: relationship between fMRI nodal parameters and top two PLS components for analysis with distance alone as response variable.

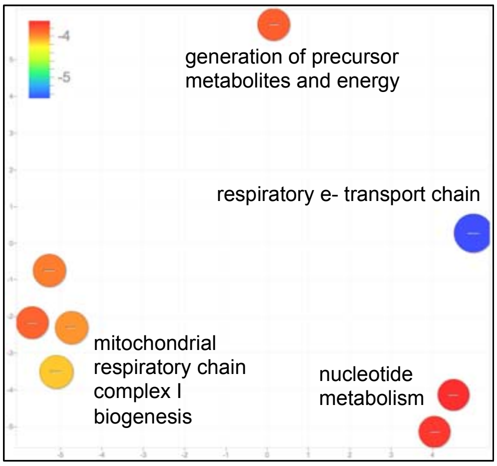

Figure S14: relationship between fMRI nodal parameters and top two PLS components for analysis with distance alone as response variable.

While the overall pattern of results is similar to that presented in the main text, we note that there is also an interesting shift in cytoarchitectonic and transcriptomic correlates of PLS2 as connection distance is more heavily weighted in the response variable. This is shown in Figure S15, which compares the PLS analysis from the main text (panel A) to that with four modules (panel B, supplementary section 5) and finally that with eight modules but using distance alone as a response variable (panel C). We see that the correlation between PLS2 scores and nodal distance increases across these cases. For panel B, this can be explained by the fact that coarser modules will restrict inter-modular links to be longer distance, while in panel C PLS2 is by definition driven exclusively by covariance with nodal distance. With this increasing weighting of long distance connectivity, we note that association cortical areas of class 3 play an increasingly important role in PLS2. In tandem, the component becomes increasingly enriched in AG genes.

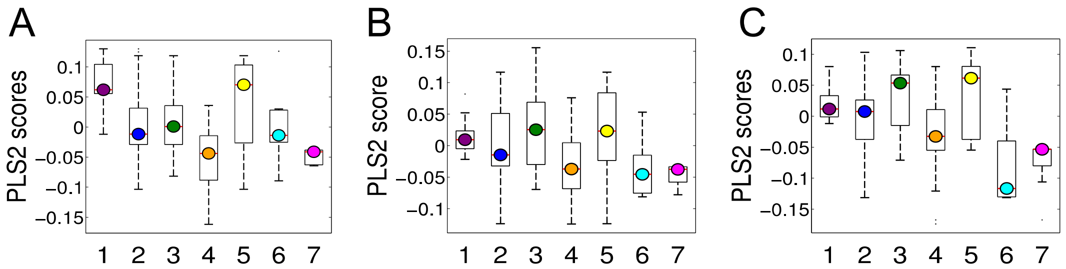

- PLS2 vs d: R=0.28
- AG gene enrichment P=0.9

- PLS2 vs d: R=0.45
- AG gene enrichment P=0.055

- PLS2 vs d: R=0.53
- AG gene enrichment P<0.001

Figure S15: Cytoarchitectonic and transcriptomic characteristics of PLS2 with increasing weighting of long distance connectivity in PLS. (A) cytoarchitectonic patterning of PLS2 for the case presented in the main text (8 fMRI modules, k_{inter}, k_{intra} and d as response variables). The panel also shows the R-value for the correlation between PLS2 and average nodal distance d, as well as the P-value for the enrichment of AG genes in PLS2 gene weights. Both of these relationships increase in significance when considering 4 fMRI modules (still with k_{inter}, k_{intra} and d as response variables) (B) and when considering 8 fMRI modules with d alone as response variable (C). Cytoarchitectonic classes are numbered and colour-coded according to the same scheme as in Figure 1 of the main text. Class1 (purple): granular cortex, primary motor cortex. Classes 2 and 3 (blue and green): association cortex. Class 4 (orange): dysgranular cortex, primary/secondary sensory cortex. Class 5 (yellow): agranular cortex, primary sensory cortex. Class 6 (cyan): limbic, allocortex. Class 7 (magenta): insular cortex.

8. Robustness of results to AIBS donor data included

In the main text, we averaged the AIBS data across all available donors to maximize signal to noise. Here we use a leave-one-out approach to show that results are not driven by an atypical donor.

In particular, supplementary file **Vertes2016_AIBSdonors_sensitivity.xlsx** shows that the top two PLS components preserve their relationships with fMRI nodal metrics and spatial coordinates (sheet 1) in all 6 analyses leaving out one of the donors. As one might expect, the pattern of results is more stable for PLS1 which explains a higher proportion of variance. Sheets 2 to 7 of the same file (named according to the donor excluded from analysis in each case) show that the PLS2 enrichment in

oxidative metabolism and mitochondrial respiration is again preserved across all analyses, although the significance level of these enrichment results is variable (but highly significant in each case).

Here too, results remained significant in each case with block-wise permutation testing ($P < 0.001$ for blocks defined by the anatomical regions of Desikan-Killiany atlas, left hemisphere only).

Finally, we note that the pattern of enrichment for the two candidate gene sets is generally preserved, such that HSE genes are enriched ($P<0.001$) in PLS2 while AG genes are not ($P>0.05$). The exception to this is when donor H0351.2002 is excluded (HSE enrichment of PLS2 $P>0.05$, AG enrichment of PLS2 $P<0.001$). We note that the subject in question was one of only two donors with samples collected across both hemispheres. This implies that the algorithm matching MRI regions of interest to AIBS sample locations will be significantly less precise when the subject is excluded. It is unclear whether the discrepancy above results from decreased quality of matching or decreased signal to noise in transcriptomic data or whether it reflects a true bias driven by this particular subject.

9. Neuroscience in Psychiatry Network (NSPN) Consortium author list

Chief investigator:

Ian Goodyer

Principal investigators:

Edward Bullmore
Raymond Dolan
Peter Fonagy
Peter B Jones

Associated faculty:

Paul Fletcher
John Suckling
Nikolaus Weiskopf
Pasco Fearon

Project managers:

Becky Inkster
Gita Prabhu

Postdoctoral research associates and associated research fellows:

David Bernal-Casas
Eran Eldar
Taposhri Ganguly
Tobias Hauser
Konstantinos Ioannidis
Gemma Lewis
Alda Mita
Michael Moutoussis

Sharon Neufeld
Ela Polek-MacDaeid
Rafael Romero-Garcia
Michelle St Clair
Roger Tait
Umar Toseeb
Anne-Laura van Harmelen
Petra Vértes
Kirstie Whitaker
Geert-Jan Will
Gabriel Ziegler
Jorge Zimbron

PhD students:

Joost Haarsma
Sian Davies
Juliet Griffin
Michael Hart
František Váša
Konrad Wagstyl

Data managers:

Cinly Ooi
Barry Widmer

Research assistants:

Ayesha Alrumaithi
Sarah Birt
Kalia Cleridou
Hina Dadabhoy
Ashlyn Firkins
Sian Granville
Elizabeth Harding
Alexandra Hopkins
Daniel Isaacs
Janchai King
Clare Knight
Danae Kokorikou
Christina Maurice
Cleo McIntosh
Jessica Memarzia
Harriet Mills
Ciara O'Donnell
Sara Pantaleone
Jennifer Scott
Alison Stribling

Administration team:

Junaid Bhatti
Neil Hubbard
Natalia Ilicheva
Michael Kentell
Ben Wallis
Laura Villis

References:

[1] H. Abdi, Partial least squares regression and projection on latent structure regression (PLS regression). Wiley Interdiscip. Rev. Comput. Stat 2, 97–106 (2010).

[2] C. F. von Economo, G. N. Koskinas, Atlas of Cytoarchitectonics of the Adult Human Cerebral Cortex. L. C. Triarhou, Ed., (Karger, Basel, 2008).
